# Low-intensity vibration restores nuclear YAP levels and acute YAP nuclear shuttling in mesenchymal stem cells subjected to simulated microgravity

**DOI:** 10.1038/s41526-020-00125-5

**Published:** 2020-12-01

**Authors:** Matthew Thompson, Kali Woods, Joshua Newberg, Julia Thom Oxford, Gunes Uzer

**Affiliations:** 1 Mechanical and Biomedical Engineering, Boise State University, Boise, ID USA; 2 Biomolecular Sciences Graduate Program, Boise State University, Boise, ID USA

**Keywords:** Biomedical engineering, Stem cells

## Abstract

Reducing the musculoskeletal deterioration that astronauts experience in microgravity requires countermeasures that can improve the effectiveness of otherwise rigorous and time-expensive exercise regimens in space. The ability of low-intensity vibrations (LIV) to activate force-responsive signaling pathways in cells suggests LIV as a potential countermeasure to improve cell responsiveness to subsequent mechanical challenge. Mechanoresponse of mesenchymal stem cells (MSC), which maintain bone-making osteoblasts, is in part controlled by the “mechanotransducer” protein YAP (Yes-associated protein), which is shuttled into the nucleus in response to cyto-mechanical forces. Here, using YAP nuclear shuttling as a measurement outcome, we tested the effect of 72 h of clinostat-induced simulated microgravity (SMG) and daily LIV application (LIV_DT_) on the YAP nuclear entry driven by either acute LIV (LIV_AT_) or Lysophosphohaditic acid (LPA), applied after the 72 h period. We hypothesized that SMG-induced impairment of acute YAP nuclear entry would be alleviated by the daily application of LIV_DT_. Results showed that while both acute LIV_AT_ and LPA treatments increased nuclear YAP entry by 50 and 87% over the basal levels in SMG-treated MSCs, nuclear YAP levels of all SMG groups were significantly lower than non-SMG controls. LIV_DT_, applied in parallel to SMG, restored the SMG-driven decrease in basal nuclear YAP to control levels as well as increased the LPA-induced but not LIV_AT_-induced YAP nuclear entry over SMG only, counterparts. These cell-level observations suggest that daily LIV treatments are a feasible countermeasure for restoring basal nuclear YAP levels and increasing the YAP nuclear shuttling in MSCs under SMG.

## Introduction

The musculoskeletal deterioration that astronauts experience on long-term space missions and the resulting increase of traumatic physical injury risk is in part due to the reduction of mechanical loading on the musculoskeleton^1^. To alleviate the detrimental effects of unloading, astronauts undergo intensive regimens of running and resistance training in orbit^2^. Despite these efforts, astronauts lose an average bone density of 1% for each month they spend in space^3^. This loss necessitates new non-pharmacologic therapies, in addition, to exercise to keep musculoskeleton healthy during long-term space missions. In bone, tissue-level response to mechanical challenge is in part regulated by osteoblasts and osteocytes^4^. Both osteoblasts and osteocytes in turn share a common progenitor: the mesenchymal stem cell (MSC). Therefore, the growth and differentiation of MCSs in response to mechanical stimulation is required for the maintenance and repair of bone^5^. It is for this reason that the MSCs are a potential target for mechanical therapies aiming to alleviate bone loss in astronauts, injured service personnel with long periods of bed rest, and physically inactive aged individuals^6^.

To maintain healthy bone-making cell populations, MSCs rely on environmental mechanical signals inside the bone marrow niches and near bone surfaces. While the exact characteristics of the mechanical environment in which MSCs exist remain to be quantified, it is known that during habitual activities, our bones are subjected to combinations of complex loads including strain, fluid shear, and acceleration, each of which is inseparable^7^. For example, during moderate running, cortical bone can experience strains up to 2000 µε^8,9^, which also generates coupled fluid flow within canaliculi of up to 100 µm/s^10^. The interior of the bone is filled with bone marrow with viscosities in the range of 400–800 cP^11^. During moderate running, tibial accelerations are within the 2–5 g range^12^ (1 g = 9.81 m/s^2^), creating a complex loading at the bone marrow interface that depends on many factors including frequency, amplitude, and viscosity^13^. In silico studies reveal that when exposed to vibrations (0.1–2 g), marrow-filled trabecular compartments generate fluid shear stresses up to 2 Pa^13,14^, capable of driving bone cell functions^15^. Interestingly, while these high magnitude forces are only experienced a few times during the day, bones are bombarded by smaller mechanical signals arising from muscle contractions that generate bone strains ranging between 2 and 10 µε^16^.

Exogenous application of these small magnitude mechanical signals in the form of low-intensity vibrations (LIV) ranging between 0.1 and 2 g acceleration magnitudes and 20–200 Hz frequencies were shown to be effective in improving the tissue level bone and muscle indices^17^. The application of LIV has been shown to be effective in preclinical and clinical studies. Animal studies demonstrate that LIV increases trabecular bone density and volume^18^, enhance bone stiffness and strength^19^, and to slow bone loss caused by disuse^20^. Further, LIV enhanced muscle contractility^21^, strength^22^, and cross-sectional area^23^, showing that LIV signals are anabolic to skeletal muscle. Clinical studies support the beneficial effects of LIV where twice-daily exposure to LIV for one year increased bone mineral density in both postmenopausal^24^ and premenopausal women^25^, LIV resulted in enhanced muscle strength, size, and function in clinical trials^25–27^. Supporting the effectiveness of LIV in 3D in vivo environments, the application of LIV in both horizontal and vertical directions result in similar effects at the cell level in vitro^28^. At the cellular level, using both horizontal and vertical LIV systems, our group has reported that the application of LIV increases MSC contractility^28^, activates RhoA signaling^29^, and results in increased osteogenic differentiation as well as increased proliferation of MSCs^30^.

The cellular response relies on sensing and intracellular transduction of environmental information. This information is either coded in the extracellular matrix as growth factors or activates mechano-sensitive signaling cascades through dynamic environmental force gradients. Molecules that shuttle between cytoplasm and nucleus in response to mechanical forces such as βcatenin and Yes-associated protein (YAP), along with its orthologue TAZ, are widely recognized as molecular “transducers” of mechanical information^17^. YAP and its paralog TAZ have important overlapping but sometimes differing functions in stem cells. Focusing on the skeletal system, the interdependent functioning of YAP and TAZ is integral in skeletogenesis and bone regeneration. Not only does the deletion of both YAP and TAZ result in skeletal deficits^31^, but they also modulate the function and expression of the master osteogenic transcription factor Runx-2 in stem cells. For example, TAZ forms complexes with Runx-2 to increase its function^32^, and TAZ nuclear presence positively drives MSC osteogenesis^32^. YAP, on the other hand, maintains stem cell multipotentiality through repressing Runx-2 function^33^ and promoting the expression of Wnt inhibitory molecule Dkk-1^34^. Further, we have shown that the absence of nuclear YAP amplifies osteogenesis in a Runx-2-dependent manner^35^. On the other hand, when YAP and TAZ enter into the nucleus, they both bind to their co-transcriptional activator TEAD and increases cell proliferation^36,37^. Therefore, while both YAP and TAZ act as pro-proliferative transcriptional co-factors for TEAD as well as playing differential role in osteogenesis, to function they need to shuttle from the cytoplasm into the cell nucleus. It has been reported that YAP nuclear entry is triggered by soluble or mechanical factors that increase F-actin contractilities such as Lysophosphatidic acid (LPA)^38,39^, increased substrate stiffness^40^, or substrate stretch ranging from 3 to 15%^41,42^, it is not known if LIV which we have shown to increase RhoA signaling^29^ and MSC contractility^28^ also trigger acute YAP nuclear entry.

The ability of molecular transducers such as β-catenin^43^, YAP, and TAZ to move between cytoplasm and nucleus has been associated with Linker of Nucleoskeleton and Cytoskeleton (LINC) complex function that physically couple cytoskeleton into nuclear lamina^44^ where polymerized F-actin binds to Nesprin (Nesprin-1 or Nesprin-2), spectrin repeat protein that pierces the nuclear envelope, connecting via its “KASH” (Klarsicht, ANC-1, Syne Homology) domain to intra-membrane leaflet Sun (Sun-1 and Sun-2) proteins^45^. When the LINC complex was de-functionalized via Nesprin deletion (siRNA), the strain was unable to impel YAP transport into the nucleus^41^. It has been further reported that pharmacologic inhibition of cytoskeletal contractility via cytochalasin D or disrupting nucleo-cytoskeletal connectivity via depletion of LINC complex function mutes the YAP nuclear entry triggered by either direct force application to nuclei via AFM or by increased substrate stiffness^44^.

The research aimed at studying the effects of microgravity at the cellular level often relies on simulated microgravity (SMG) devices designed to alter the gravitational conditions that cells experience by rotating on one or multiple axes at low speed^46–48^. Clinostats with a monolayer of cells is a microgravity model system^49^ and it has been used in a number of scientific studies to model aspects of microgravity since 70 s^47,50–52^. SMG decreases MSC proliferation^53^ and cytoskeletal contractility^46,54,55^. While SMG was reported to decrease nuclear TAZ levels^52^, the role of SMG on nuclear YAP levels is unknown.

As SMG effects are commonly associated with “unloading”, application of physical or soluble factors that induce cytoskeletal contractility are frequently used as countermeasures for SMG^51,52^. In this way, LIV was previously shown to improve decreased osteogenesis of preosteoblasts under SMG^56^. We have recently reported that SMG decreases cell proliferation and total YAP protein levels while daily LIV application can alleviate the decreased proliferation and YAP protein levels in MSCs^57^. This LIV effect was dependent on LINC complex function as depleting Sun-1 and Sun-2 element of the LINC complex muted the LIV response. Importantly, we have also reported that SMG decreases nuclear Lamina element LaminA/C as well as the Sun-2 element of the LINC complex, which was only partially recovered by daily LIV application. Sun-1 and Sun-2 were shown to have a role in maintaining nuclear YAP levels^58^ and Sun-2 was shown to interacts with F-actin cytoskeleton to maintain YAP nuclear entry in response to strain^59^. In this way, YAP-mechanosignaling may be altered by SMG due to alterations to the nuclear envelope. This suggests the possibility that LIV can be a feasible SMG countermeasure for increasing the YAP nuclear shuttling in response to subsequent mechanical challenges or soluble activators such as LPA.

Therefore, we tested whether SMG reduces basal nuclear YAP levels as well as YAP nuclear shuttling driven by either acute LIV (LIV_AT_) or Lysophosphohaditic acid (LPA), applied at the end of the 72 h period SMG period. We have further tested if daily LIV treatment (LIV_DT_), which was applied in parallel to SMG during the 72 h period alleviates SMG-driven effects on basal nuclear YAP levels as well as acute YAP nuclear shuttling driven by either acute LIV_AT_ or LPA. We hypothesized that SMG-induced impairment of basal nuclear YAP levels as well as YAP nuclear entry in response to LIV_AT_ and LPA would be alleviated by the daily application of LIV_DT_.

## Results

### Acute LIV_AT_ application increases nuclear YAP levels

**Fig. 1:**
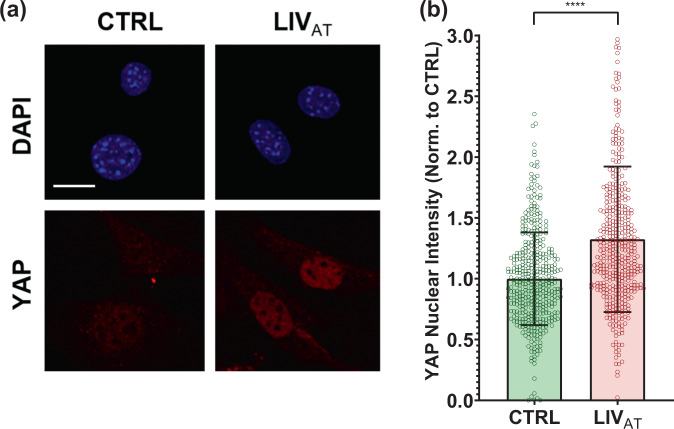
Acute LIV_AT_ application increases nuclear YAP levels. **a** MSCs were subjected to LIV_AT_ and stained with DAPI (blue) and YAP (red). Confocal images indicated increased nuclear YAP levels following acute LIV_AT_ applied as five 20 min vibration periods separated by 1 h. **b** Quantitative analysis of confocal images showed a 32% increase of nuclear YAP in LIV_AT_ samples compared to controls. *n* > 400/grp, group comparison was made a Mann–Whitney *U*-test, ****p < 0.0001. Error bars represent standard deviation. Scale bar: 10 μm. Full statistical details were provided in Supplementary Table 4.

To quantify the acute YAP nuclear entry in response to LIV, MSCs were plated at a density of 5200 cells/cm^2^ and were allowed to attach for 24 hr. Following this, MSCs were subjected to treatment in two groups: control and acute LIV treatment regimen (LIV_AT_). The LIV_AT_ regimen consisted of 5 × 20 min vibration periods separated by 1 hr in between each repetition at room temperature while control samples were treated identically (also taken out of the incubator) but were not vibrated. Immediately after LIV_AT_, samples were immunostained for YAP and DAPI. MATLAB was used to quantify the changes in the nuclear YAP levels. As shown in Fig. 1a, confocal images showed increased nuclear YAP following the LIV_AT_ treatment. For this and all subsequent experiments, the average pixel intensities for all imaged nuclei per sample were normalized to the mean of all the nuclei for the control sample and then presented on a bar graph in order to compare average protein concentration in arbitrary units. Analysis of confocal images to quantify nuclear YAP intensity shown in Fig. 1b revealed a 32% increase in the average nuclear YAP levels in the LIV_AT_ samples as compared to the control samples (*p* < 0.0001). We also used C2C12 myoblasts to confirm the LIV_AT_-induced YAP nuclear entry on a second cell line, quantitative analysis of confocal images showed a 40% increase of nuclear YAP in LIV_AT_ samples compared to controls (Supplementary Fig. 1). As both LIV-induced focal adhesion signaling, initiated by focal adhesion kinase (FAK) phosphorylation at Tyr 397 residue^29^, and YAP nuclear entry in response to substrate strain^41^ requires intact LINC function, disabling LINC function via a dominant-negative overexpression of Nesprin KASH (Klarsicht, ANC-1, Syne homology) fragment both decreased basal nuclear YAP levels by 34% (*p* < 0.0001) and impeded the LIV-induced YAP nuclear entry when compared to empty plasmid (Supplementary Fig. 2). FAK phosphorylation at Tyr 397 residue (pFAK) was blocked via a FAK inhibitor (FAKi) PF573228 (3 µM) 1 h prior to LIV_AT_ treatment as previously described^29^ and stained against DAPI and YAP (Supplementary Fig. 3a). FAKi inhibited the LIV_AT_-induced pFAK and decreased its basal levels (Supplementary Fig. 3b). As shown in Supplementary Fig. 3c, measuring nuclear YAP levels showed that LIV_AT_-induced YAP nuclear entry was not affected by FAKi when compared to DMSO-treated controls.

### Basal nuclear YAP levels decreased by SMG were rescued by daily application of LIV_DT_

**Fig. 2:**
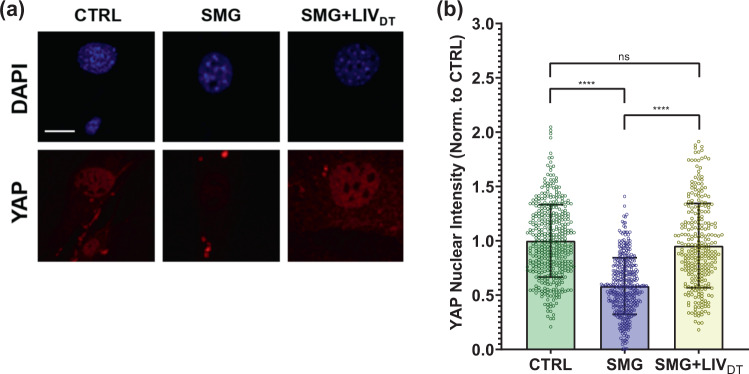
Basal nuclear YAP levels decreased by SMG were rescued by LIV_DT_. **a** MSCs were subjected to SMG, and SMG + LIV_DT_ over 72 h period and stained with DAPI (blue) and YAP (red). **b** Quantitative analysis showed a 42% decrease in nuclear YAP levels in the SMG group compared to control levels. The SMG + LIV_DT_ group showed a 67% increase in nuclear YAP when compared to the SMG group. There was no statistically significant difference between CTRL and SMG + LIV_DT_ groups. *n* > 100/grp. Group comparisons were made via Kruskal–Wallis test followed by Tukey multiple comparison, ****p < 0.0001. Error bars represent standard deviation. Scale bar: 10 μm. Full statistical details were provided in Supplementary Table 5.

We next tested whether SMG decreases basal YAP levels and whether a daily LIV treatment regimen (LIV_DT_), applied in parallel with SMG, could alleviate decreased YAP in the nucleus. As we reported previously, LIV_DT_ consisting of 2 × 20 min vibrations applied every 24 h during the 72 h period of SMG. This LIV_DT_ regimen was effective at restoring MSC proliferation and whole-cell YAP levels when applied in conjunction with SMG^57^. MSCs were plated at a density of 1700 cells/cm^2^ in 9 cm^2^ tissue culture SlideFlasks (Nunc, #170920) and were allowed to attach for 24 h, after which point, the flasks were filled completely with growth medium, sealed, and subjected to 72 h of treatment followed by immunostaining for YAP and nuclear staining using DAPI. During the 72 h treatment period, MSCs were divided into three groups: control samples, SMG samples, which were subjected to the 72 h SMG alone, and SMG + LIV_DT_ samples, which were subjected to both the 72 h SMG regimen and the daily LIV_DT_ regimen. Representative images for YAP and DAPI stained images are shown in Fig. 2a. As depicted in Fig. 2b, the quantitative analysis of confocal images revealed a 42% decrease in the nuclear YAP intensity of the SMG group as compared to non-SMG controls (*p* < 0.0001). Compared to the SMG group, the LIV_DT_ group increased nuclear YAP levels by 67% (*p* < 0.0001), and there was no significant difference between nuclear YAP levels of LIV_DT_-treated MSCs and non-SMG controls.

### LIV_AT_-induced YAP nuclear entry decreased by SMG was not restored by daily LIV_DT_ application

**Fig. 3:**
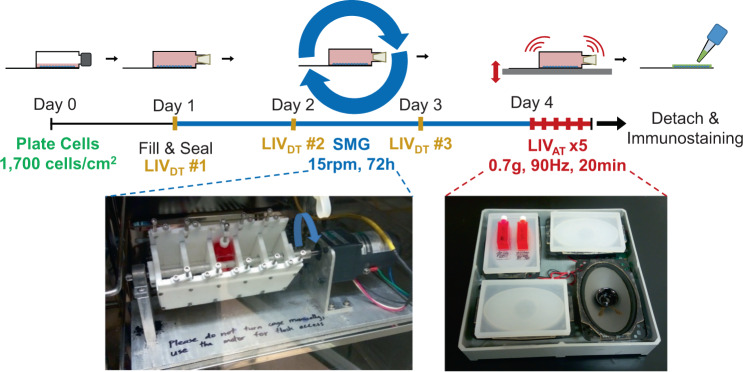
Experimental design of combined SMG, LIV_AT_, and LIV_DT_ application. MSCs were subcultured and plated in SlideFlasks and allowed to attach for 24 h before SlideFlasks were filled with culture medium and sealed. The treatment regimen for MSC’s involved 72 h of SMG (blue). LIV_DT_ regimen consisted of one treatment cycle every 24 h during SMG treatment with each cycle consisting of 2 × 20 min LIV with an hour in between (yellow). LIV_AT_ regimen was applied after 72 h SMG treatment period and consisted of 5 × 20 min LIV with an hour in between each (red). For LIV application, MSCs plated in SlideFlasks were placed in an LIV device constructed in the lab previous to this research. Vibrations were applied at peak magnitudes of 0.7 g at 90 Hz at room temperature. Control samples were treated the same but were not vibrated. For SMG application, MSCs plated in SlideFlasks were secured in lab custom-built clinostat inside the incubator. The clinostat subjected the MSCs to constant 15 RPM rotation simulated microgravity. After treatment, flasks were removed for immunofluorescence staining.

**Fig. 4:**
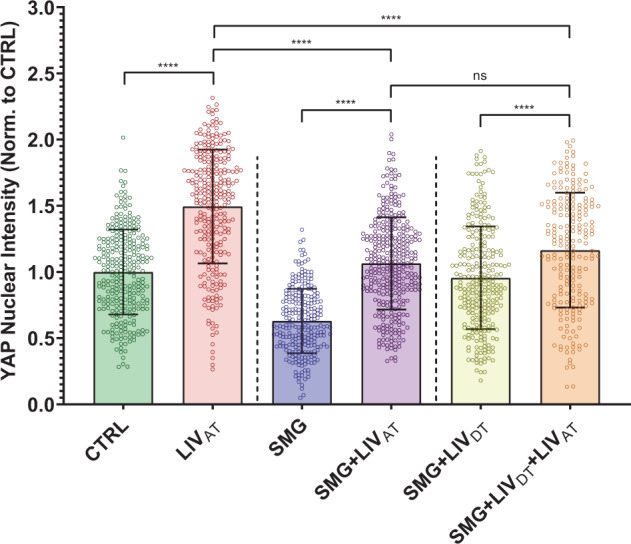
LIV_AT_-induced YAP nuclear entry decreased by SMG was not restored by daily LIV_DT_ application. MSCs were subjected to either CTRL, SMG, SMG + LIV_DT_ over 72 h period were subsequently treated with LIV_AT_. Quantitative analysis of confocal images showed that SMG alone decreased basal nuclear YAP levels by 37%, which were increased back to control levels in the SMG + LIV_DT_ group. +LIV_AT_ increased nuclear YAP levels in the CTRL, SMG, and SMG + LIV_DT_ groups by 50%, 69%, and 22%, respectively. Nuclear YAP intensity in the SMG + LIV_DT_ + LIV_AT_ group remained not significantly different from the SMG + LIV_AT_ and 23% smaller than the LIV_AT_ group. *n* > 200/grp. Group comparisons were made via Kruskal–Wallis test followed by Tukey multiple comparison, ****p < 0.0001. Error bars represent standard deviation. Scale bar: 10 μm. Full statistical details were provided in Supplementary Table 6.

As SMG decreased basal nuclear YAP levels, we next tested whether SMG decreases LIV_AT_-induced YAP nuclear shuttling and whether LIV_DT_ application alleviates this. A schematic of the experimental design is given in Fig. 3. MSCs were divided into six groups in which the CTRL, SMG, SMG + LIV_DT_ groups were treated with ±LIV_AT_ at the end of 72 h and nuclear YAP levels were measured. As shown in Fig. 4, SMG alone decreased basal nuclear YAP levels by 37% (*p* < 0.0001), which were restored back to control levels in the SMG + LIV_DT_ group. As depicted in Fig. 4, +LIV_AT_ increased nuclear YAP levels in the CTRL, SMG, and SMG + LIV_DT_ groups by 50%, 69%, and 22%, respectively (*p* < 0.0001) while exhibiting the smallest increase in the SMG + LIV_DT_. As a result, final nuclear YAP levels in the SMG + LIV_DT_ + LIV_AT_ group were not significantly different from the SMG + LIV_AT_ and 23% lower than the LIV_AT_ group (*p* < 0.0001). Representative confocal images are presented in Supplementary Fig. 4.

### LPA treatment increases nuclear YAP levels

**Fig. 5:**
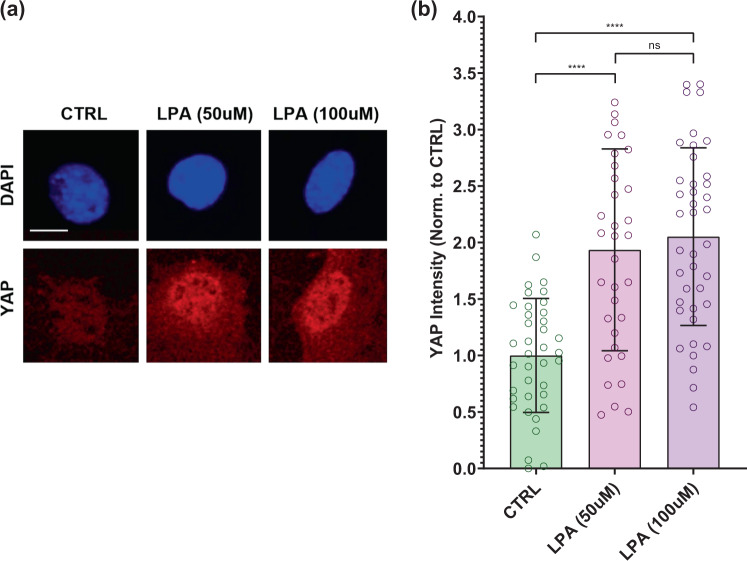
LPA treatment increases nuclear YAP levels. **a** Representative confocal images of DAPI (blue) and YAP (red) stained MSCs with or without LPA treatment. MSCs were subjected to LPA addition at 50 and 100 µM concentrations. **b** Quantitative analysis of confocal images revealed a 99% and a 107% increase in the 50 µM LPA and 100 µM LPA treatments compared to DMSO-treated controls, respectively. Nuclear YAP levels for 50 µM LPA and 100 µM LPA treatments were not significantly different. *n* > 30/grp. Group comparisons were made via Kruskal–Wallis test followed by Tukey multiple comparison, ****p < 0.0001. Error bars represent standard deviation. Scale bar: 10 μm. Full statistical details were provided in Supplementary Table 7.

As the lack of difference between SMG + LIV_AT_ and SMG + LIV_DT_ + LIV_AT_ treatment (Fig. 4) suggested that dosing cells daily with LIV_DT_ may decrease cell responsiveness to LIV-AT, we sought an alternative soluble activator of YAP nuclear shuttling. LPA is frequently used for activating RhoA-mediated cytoskeletal response^60,61^. We have previously shown that the LPA effect on F-actin cytoskeleton and focal adhesion activation is additive with both LIV and substrate strain^29^. Since the usage of sealed culture flasks did not permit us to use substrate strain and LPA has been shown to induce nuclear YAP-shuttling in a number of cell types^38,39,62,63^, we utilized LPA as a soluble activator of YAP signaling. To test the effect of LPA on the acute YAP nuclear entry in MSCs, two LPA concentrations (50 and 100 µM) were compared against control samples. As shown in Fig. 5, nuclear YAP levels were almost doubled under a 2-h exposure to 50 µM LPA and 100 µM LPA treatments with 99 and 107% increases as compared to the control samples (*p* < 0.0001). Nuclear YAP levels for 50 µM LPA and 100 µM LPA treatments were not significantly different. Therefore, we chose to use 50 µM LPA treatment in the subsequent experiments.

### LPA-induced YAP nuclear entry decreased by SMG was alleviated by daily LIV_DT_ application

**Fig. 6:**
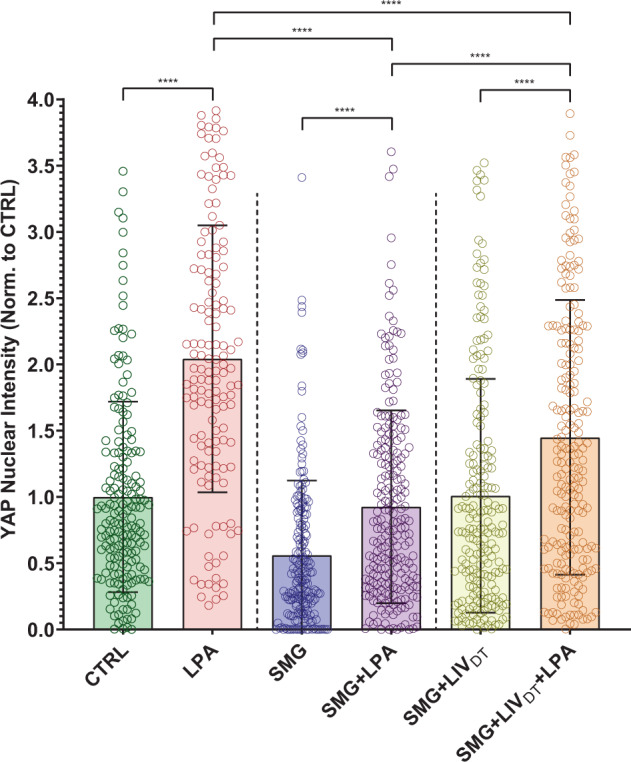
LPA-induced YAP nuclear entry decreased by SMG was alleviated by daily LIV_DT_ application. MSCs were subjected to SMG, and parallel SMG + LIV_DT_ over 72 h period at the end of 72 h, samples were treated with either LPA (50 µM) or DMSO. Quantitative analysis of confocal images revealed that LPA addition increased nuclear YAP levels by 105%, 67%, and 43% in the CTRL, SMG, and SMG + LIV_DT_ when compared to DMSO controls. When compared to nuclear YAP intensity of the LPA treatment alone, SMG + LPA and SMG + LIV_DT_ + LPA samples were 55% and 29% lower, respectively. YAP nuclear levels in SMG + LIV_DT_ + LPA remained 70% higher than SMG + LPA group. *n* > 100/grp. Group comparisons were made via Kruskal–Wallis test followed by Tukey multiple comparison, ****p < 0.0001. Error bars represent standard deviation. Scale bar: 10 μm. Full statistical details were provided in Supplementary Tables 8.

After establishing that LPA induces YAP nuclear entry in MSCs, we next evaluated whether SMG decreases LPA-induced YAP nuclear shuttling and whether LIV_DT_ application alleviates this. A 50 µM LPA dissolved in DMSO or DMSO as vehicle control was added to the samples at the end of the 72 h treatment of either CTRL, SMG, or SMG + LIV_DT_ treatments. The CTRL group, SMG group, and SMG + LIV_DT_ group were subjected to the same treatment as in the previous experiments and displayed similar results. As depicted in Fig. 6, +LPA increased nuclear YAP levels in the CTRL, SMG, and SMG + LIV_DT_ groups by 105%, 67%, and 43% respectively (*p* < 0.0001). While final YAP nuclear levels in SMG + LIV_DT_ + LPA remained 70% higher than SMG + LPA group (*p* < 0.0001), it remained 29% lower than the LPA group (*p* < 0.0001).

### MSC stiffness and structure remain intact under SMG and SMG + LIV_DT_ treatments

**Fig. 7:**
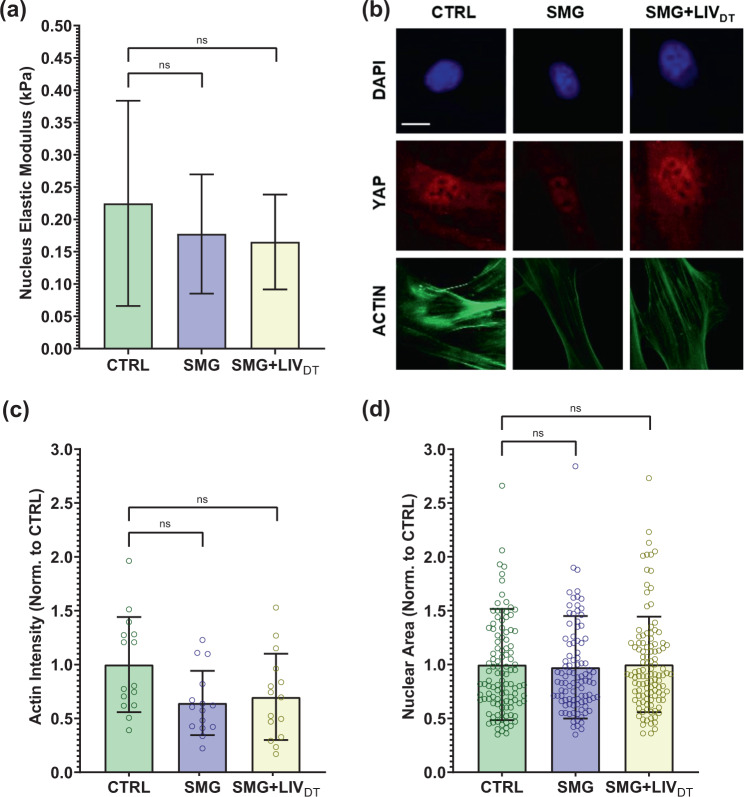
MSC stiffness and structure remain intact under SMG and SMG + LIV_DT_ treatments. MSCs were subjected to SMG and parallel SMG + LIV_DT_ over a 72 h period. **a** Compared to CTRL samples, AFM measurement of the elastic moduli of SMG and SMG + LIV_DT_-treated MSCs revealed apparent decreases in elastic modules that were 21 and 27% below control levels, measured differences were not statistically significant. *n* = 10/grp. **b** Quantification of confocal images shows that **c** mean F-actin intensity of SMG and SMG + LIV_DT_-treated MSCs revealed a decrease of 36 and 30% below control levels, measured differences were not statistically significant. *n* = 15/grp. **d** No significant effects of either SMG or LIV_DT_ treatment on the average nucleus size were found. *n* > 100/grp. Group comparisons were made via Kruskal–Wallis test followed by Tukey multiple comparison. Error bars represent standard deviation. Scale bar: 10 μm. Full statistical details were provided in Supplementary Tables 9–11.

As YAP-mechanosignaling of SMG + LIV_DT_ MSCs remained below control levels in response to both LIV_AT_ and LPA, we quantified the effects of SMG and SMG + LIV_DT_ on the cell stiffness, F-actin intensity, cell area, and nuclear area. AFM testing was used to quantify the elastic modulus of the nucleus by measuring load-displacement curves on top of the nucleus. AFM tests shown in Fig. 7a indicated a 21 and 27% stiffness decrease in the SMG or SMG + LIV_DT_ groups but differences were not significant. Quantified from confocal images (Fig. 7b), mean F-actin intensities for all the cells in each imaging field were quantified by dividing the mean F-actin intensity to the number of nuclei in each imaging field. Shown in Fig. 7c, SMG and SMG + LIVDT-treated MSCs revealed 36% and 30% decreases in the mean F-actin intensity per cell, respectively, but the differences were not statistically significant. We have further quantified the nuclear area as a measure of cyto-mechanical forces on the nucleus^64^. As shown in Fig. 7d, analysis of the cross-sectional area of cell nuclei using DAPI stained images revealed no significant effect on average nuclear size by either SMG or combined SMG + LIV_DT_ treatment compared to control levels.

## Discussion

The mechanical forces that the bone and muscle cells are subjected to on Earth and in microgravity are complex and remain incompletely understood. At the same time, it is clear that these forces are required for healthy tissue growth and function. The complexity of these forces makes it difficult to design experiments that comprehensively simulate in vivo conditions. While, spheroid systems that provide in vivo like 3D growth conditions^65^, spheroid systems are harder to design in terms of fluid shear. Earlier studies that consider cell suspension systems^66^ indicated that very low fluid shear can be achieved if cell motion within the container can be avoided (i.e., low rpm, small particle size, high viscosity, etc). However, motion tracking studies with 3D microspheres with higher-than-water and lower-than water densities showed that while less dense scaffolds avoid repeated collisions with the bioreactor wall that impose a variety of confounding, non-quantifiable mechanical disruptions, both types generated fluid shear values ranging between 0.16 and 0.32 Pa^67^. As a result, rotated groups showed increased osteogenic differential compared to static ones. In contrast, rotation devices with adherent cells do not generate as high fluid shear values. For example, maximum fluid shear at flask walls during 15 rpm rotation was found to be 0.06 Pa^68^, which order or magnitude lower compared to microbead systems. These findings led us to adopt a monolayer SMG system. Another important discussion is the fluid shear generated by vibration stimuli. Sealed flasks would largely eliminate this fluid shear and sealing culture plates and flasks to avoid fluid motion is commonly utilized in both LIV^69,70^ and sMG-based studies^48,53,71,72^. Fluid shear generated therefore will be independent of fluid volume and is a result of deformations of a fluid-bounded well-bottom due to vertical accelerations^73^. These dynamic accelerations result in lateral fluid motion over-attached cells. We have reported that vertical 0.7 g, 90 Hz LIV in 6-well culture plates results in a peak velocity difference of 0.00004 m/s between fluid and well-bottom^29^, corresponding to peak fluid shear of 0.0008 Pa, well below of SMG-induced fluid shear. In this current study, we have utilized 10 cm^2^ culture flasks, which have a 5% more surface area compared to 6-well plates (9.5 cm^2^). While this difference in the area may result in larger peak deformations and thus generate larger fluid shear, we believe that using 10 cm^2^ flasks would be to not be radically different than 6-well culture plates in terms of fluid shear. We expect these fluid shear values to be inconsequential to LIV response as we previously tested the possible contribution of fluid shear to LIV vibration response^74^. Increasing fluid shear up to 2 Pa under 100 Hz horizontal vibration did not generate a statistically significant effect when compared to the LIV stimulus that generated 0.015 Pa fluid shear. As computational and ex vivo studies suggest that LIV may generate fluid shear forces between 0.5 and 2 Pa in vivo^7^. Our findings suggest that fluid shear does not contribute to the LIV vibration response. Therefore the in vitro experiments utilizing SMG and LIV treatments used in this study are limited in replicating the in vivo conditions and do not entirely correlate with the physiological behavior of these cells in vivo, the experiments presented here remain useful for testing cell behavior under these well-defined conditions.

Another limitation of the study includes our inability to apply SMG and LIV treatments simultaneously. When daily LIV was combined with SMG, the culture flasks needed to be removed from the clinostat SMG device in order to put them in the LIV device. This limitation is primarily due to our inability to dampen vibrations in the cell culture incubator. When we turn on the vibrating device inside the cell culture incubator, all the samples, including the controls also experience significant amounts of vibration. Further, vibrating the entire SMG system (i.e., simultaneous application) is a more complex problem due to (i) weight of the SMG setup necessitates a newer LIV device design and (ii) potential structural response of SMG device to 0.7 g, 90 Hz vibrations that would generate secondary vibrations were unknown. To avoid these confounding factors, we apply LIV on an isolated table while controls sit on a separate table with no vibration signal. While disruptions to SMG cannot be avoided, between the 20 min LIV sessions for the daily LIV treatment, the flasks were placed back into the clinostat such that the interruptions in SMG treatment were brief relative to the 72 h application period. This was not done for the acute LIV treatment as this treatment was designed to test acute YAP signaling activation after 72 h of SMG.

In this study, we focused on YAP-mechanosignaling of MSCs. The first experiments demonstrated that repeated LIV_AT_ application over six hours was capable of stimulating YAP entry into the nucleus in both MSCs (Fig. 1) and in the C2C12 cell line (Supplementary Fig. 1). These findings suggest that similar to high magnitude substrate strains^42^ smaller mechanical signals such as LIV can be effective at increasing nuclear YAP levels. Further, in agreement with earlier reports utilizing uniaxial strain^41^, LIV_AT_-induced increase in nuclear YAP levels also required functional LINC complexes (Supplementary Fig. 2). Integrin related FAK signaling has been shown to promote YAP nuclear levels in the proliferative descendants of stem cells and that FAK inhibitor PF573228 decreased nuclear YAP in these cells^75^. Similarly, inhibiting integrin engagement via blocking FAK phosphorylation in Tyr 397 residue via FAKi also mutes the increase of GTP-bound RhoA levels in LIV-treated MSCs^29^. While we confirmed the loss of phosphorylation in Tyr 397 at both basal levels and in response to LIV_AT_ (Supplementary Fig. 3b), FAKi treatment changed neither basal levels nor the LIV_AT_-induced increase in nuclear YAP (Supplementary Fig. 3c), suggesting a FAK independent mechanism. In these experiments, we did not compare LIV_AT_ with strain because the application of 5–15% stretch onto sealed culture flasks was not technically possible without significantly altering experimental conditions. Instead, LPA addition served as the best option for applying a simple mechanical stimulation in order to evaluate the YAP mechanotransduction. LPA is a phospholipid derivative signaling molecule, which is capable of causing the simulation of a static transient stretch of a cell by activating GTPase Rho-mediated actin stress fiber creation, which results in increased contractility of the cytoskeleton^30,60^. The first experiments with LPA served to verify that the simulation of stretch via increased cytoskeleton contractility was capable of triggering YAP entry into the nucleus and the analysis methods utilized here were capable of detecting this response (Fig. 5).

The first SMG experiments confirmed a clear decrease of basal nuclear YAP levels. Interestingly, SMG-treated cells remained responsive, as both LIV_AT_ and LPA treatments were able to increase the nuclear YAP levels at the end of the acute stimulation period (<6 h). However, final nuclear YAP levels in SMG-treated MSCs remained significantly lower when compared to non-SMG groups (Figs. 2, 4, and 6). These findings suggested that the YAP-mechanosignaling apparatus of MSCs, to some extent, was intact under SMG. When applied in parallel to SMG, daily LIV_DT_ treatment was able to restore basal YAP levels in the cell nucleus (Figs. 2, 4, and 6) measured 24 h after the final LIV_DT_ treatment. This increase in nuclear levels supported our earlier report that showed sustained recovery of MSC proliferation by LIV_DT_^57^. An interesting observation is that a closer look into Figs. 1b, 2b, and Supplementary Fig. S1 reveals that while all the sample sets fit into a general expectation of a “bell shaped” distribution, the shape of the LIV_AT_ samples looks more unbalanced towards the positive end compared to others. The first interesting observation is that LIV_DT_ groups do not show this shift probably because the imaging was conducted 24 h after the last LIV_DT_ treatment to avoid any residual acute effects of LIV_DT_ on the nuclear YAP levels. This timing should be sufficient as nuclear YAP levels have been shown to return to baseline within 12 h^42^. Therefore, in the lieu of our previous findings that LIV_DT_ restores overall YAP levels decreased by SMG^57^, here we are seeing that nuclear YAP levels are also restored by LIV_DT_. As for the shape changes under LIV_AT_, there could be a number of reasons. First, it is possible that some cells are more responsive to LIV_AT_ than others. More importantly, we apply LIV_AT_ in five different bouts spanning a 6 h period, as YAP response to strain shown to be maximized around 6 h^42^ it may be possible that the shape is representative of the MSCs responding to LIV_AT_ at different times. In this way, it is conceivable that shape might look different if a time point different than 6 h was selected. Ultimately, while shape may represent the dynamic and acute nature of the LIV_AT_ response of YAP, the upward shift of the population intensity and the increased averages suggest increased nuclear YAP intensity in the cell nuclei under LIV_AT_ treatment_._

We next aimed to determine the effects of SMG and LIV on YAP signaling response to acute mechanical stimulus including the LIV_AT_ treatment as well as the addition of soluble LPA. Interestingly, this increase in basal nuclear YAP levels under LIV_DT_ was accompanied by a reduced MSC response to LIV_AT_ treatment (Fig. 4). When SMG + LIV_DT_-treated MSCs were subjected to LIV_AT_, the increase in nuclear YAP from the non-LIV_AT_ control was only 22% (*p* < 0.0001), which was small compared to the 77% increase seen in the SMG groups in response to LIV_AT_. As a result of this smaller increase in the SMG + LIV_DT_ group, there was no measurable difference between SMG and SMG + LIV_DT_, samples that were subjected to LIV_AT_. It has been previously reported that an application of multiple LIV bouts separated by a refractory period is more effective at activating mechanosignaling pathways such as β-catenin^76^. It is possible that long-term application of LIV_DT_ results in cell structural adaptations that serve to reduce MSC responsiveness to LIV_AT_ treatment. To test this possibility, we replaced LIV_AT_ with an LPA treatment. When LIV_AT_ was replaced by LPA treatment (Fig. 6), the responsiveness of SMG + LIV_DT_-treated MSCs almost doubled to 43% (compared to 22% in response to LIV_AT_) and was significantly higher than the SMG + LPA group (*p* < 0.0001), suggesting that LIV_DT_ increases the YAP-mechanosignaling in response to LPA.

Absolute nuclear YAP intensity in the SMG + LIV_DT_ + LIV_AT_ group, however, remained below the LIV_AT_ group (*p* < 0.0001). Previously published findings using the same treatment protocols suggested that the total cellular YAP levels decreased by SMG were restored to control levels by daily LIV^57^. This indicates that the total availability of YAP protein was not responsible for this difference between the SMG + LIV_DT_ + LIV_AT_ and the LIV_AT_ groups. In regards to other potential effects of SMG on the components of the mechanosignaling mechanism, one current prevailing hypothesis suggests a role for nuclear pore opening in response to cyto-mechanical forces^44^, which may be affected by changes in the nuclear stiffness. To test this possibility, we performed additional AFM and imaging experiments. While the AFM measured nuclear stiffness was 24% lower in the SMG and SMG + LIV_DT_ groups on average, we were unable to identify any statistically significant effects of SMG or LIV_DT_ treatment on nuclear stiffness. There was also slight F-actin intensity decreases in both the SMG and SMG + LIV_DT_ groups, which were also not significant (Fig. 7). Similarly, cell and nuclear areas were not affected. While our results were not able to detect any changes in nuclear stiffness, considering the significant role that the nuclear membrane has as a mechanical structural component in the cell’s interpretation of mechanical stimulus^77,78^, more detailed future studies are needed to study the effects of SMG on the nuclear envelope and nuclear structure.

In summary, while the restoration of basal nuclear levels and improvement of LPA-induced YAP nuclear entry under daily LIV_DT_ treatment identify LIV as a possible countermeasure to improve YAP nuclear import under simulated microgravity, future studies are required to understand why acute YAP nuclear entry in response LIV_AT_ remains less responsive. Additionally, restoration of basal nuclear YAP under SMG + LIV_DT_ may explain the previously observed increase in cell proliferation, the current study scope was limited to measuring basal YAP levels and YAP-shuttling only. Therefore, how these basal YAP levels and YAP-shuttling differences may affect MSC response to subsequent signaling, including proliferation and osteogenesis will be important to address in future studies to establish LIV as a viable countermeasure for unloading and microgravity.

## Methods and materials

### Cell culture

The primary mouse MSCs were isolated from multiple mice donors (8–10-wk-old male B6 mice)^29,43,57^. Briefly, these primary MSCs were extracted from 3 to 5 mice and all the cells were pooled together. Following the extraction protocol^79^, these MSCs were tested for adipogenic as well as osteogenic potential and subsequently frozen. For these experiments, MSCs between passages 6 and 9 were used. We have recently reported using the same protocol that these MSCs retain their multipotentiality beyond passage 15 as shown by unaltered expression of different lineage markers such as FGF-2. RUNX-2, PPARG, SOX-2, and SOX-2^80^. C2C12 mouse myoblasts were derived from muscle satellite cells. MSCs were subcultured and plated in Iscove modified Dulbecco’s cell culture medium (IMDM, 12440053, Gibco) with 10% fetal calf serum (FCS, S11950H, Atlanta Biologicals) 10,000 unit/mL penicillin, and 10,000 μg/mL streptomycin (referred as 1% pen/strep). C2C12s were subcultured and plated in Dulbecco’s modified Eagle’s medium (DMEM, DML09, Caisson Laboratories) with 10% fetal calf serum (FCS, S11950H, Atlanta Biologicals) and 1% pen/strep. For each passage, stock cells were seeded in 55 cm^2^ culture dishes at a density of 1000 cells/cm^2^ and subcultured after an average of five population doublings at an average density of 32,000 cells/cm^2^. For experiments, MSCs were plated in SlideFlasks (Nunc, #170920) at a density of 5200 cells/cm^2^ for 1-day experiments and 1700 cells/cm^2^ for 3day experiments, while C2C12s were plated at a density of 10,000 cells/cm^2^. Experimental cells were plated and given 24 h to attach to the mounting surface prior to experiments. Cell passages for both MSCs and C2C12s used for experiments were limited to P7-P15. All methods were carried out in accordance with relevant guidelines and regulations of the Boise Institutional Animal Care and Use Committee and Institutional Biosafety Committee. All procedures were approved by the Boise State University Institutional Animal Care and Use Committee, and the Institutional Biosafety Committee.

### Low-intensity vibrations treatment

SlideFlasks with plated MSCs were filled completely with the culture medium and placed in an LIV device designed and used in our lab (Fig. 3)^57^. LIV device subjected cells to low intensity 90 Hz lateral vibrations at 0.7 g at room temperature. MSCs were vibrated for 20 min intervals separated over time. The daily LIV_DT_ regimen consisted of treatment cycles consisting of 2 × 20 min LIV with a 2 h refractory period in between, with one such treatment cycle applied per day over 3 days or 72 h. The acute LIV_AT_ regimen consisted of 5 × 20 min LIV treatments with an hour in between each. Control samples were subject to the same growth conditions except they were not placed in the LIV device and were placed on a separate table surface entirely to isolate the cells from vibrations.

### Simulated microgravity treatment

SlideFlasks (Nunc, #170920) with plated MSCs were filled completely with the culture medium and placed in a clinostat SMG device (Fig. 3). The clinostat shown is a redesign of a custom-made clinostat constructed in our lab with a new flask holder casing capable of holding SlideFlasks and fabricated with PTFE, which could be thoroughly sanitized by autoclaving The clinostat simulated microgravity by rotating the MSC’s in SlideFlasks at a constant 15 RPM, effectively rotating in one dimension the gravity vector acting on the sample and canceling out its effects as a result of the averaging out of the opposing gravity vectors over time. In all treatments, the clinostat was used to subject the MSCs to constant 15 RPM SMG for 72 h. When the LIV_DT_ regimen was combined with this treatment, the design of the clinostat and design of the LIV device required that the SlideFlasks be removed from the clinostat and placed into the LIV device for the 20 min LIV treatment intervals. However, the SlideFlasks would be placed back into the clinostat for the full duration of the 2 h refractory periods. Additionally, when the LIV_DT_ regimen was combined with this treatment, the samples that were subjected to control and SMG treatment were also taken out of the clinostat and placed on a separate table surface entirely to isolate the cells from vibrations. The LIV_AT_ regimen, on the other hand, was only ever applied after SMG treatment and therefore the SlideFlasks were not placed back into the clinostat between LIV treatments.

### Immunofluorescence staining and image analysis

Immediately after mechanical treatment, MSCs plated in Slideflasks were removed from treatment, and the SlideFlasks were disassembled in order to stain the MSCs on the slides (Fig. 3). The MSCs were fixed with 4% paraformaldehyde, then washed and permeabilized with 0.05% Triton X-100 in PBS, followed by immunostaining with YAP-specific antibody (YAP, D8H1X) Rabbit mAb, Cell Signaling Technologies #14074 at 1/100 dilution in PBS) and Alexa Fluor red secondary antibodies (Goat anti-Rabbit IgG (H + L) Cross-Adsorbed Secondary Antibody Alexa Fluor Plus 594, Thermo Fisher Scientific #A11037 at 1/500 dilution in PBS was used for all experiments prior to usage of LPA. Subsequently, Goat anti-Rabbit IgG (H + L) Cross-Adsorbed Secondary Antibody Alexa Fluor Plus 633, Thermo Fisher Scientific #A21070 at 1/500 dilution in PBS was used). Nuclear DNA was labeled via DAPI (VECTASHIELD HardSet Antifade Mounting Medium with DAPI, Vector Laboratories #H1500). Actin filaments were labeled via phalloidin stain (Phalloidin-iFluor 488 Reagent, Abcam #AB176753 at 1/500 dilution in PBS). Stained samples were imaged with a Leica TCS SP8 confocal microscope (×40x, HC PL APO CS2 Oil Immersion) prior to usage of LPA, after this point in time, the Leica machine became inoperable and therefore a Zeiss LSM 510 Meta Confocal Microscope (40x, HC PL APO CS2 Oil Immersion) was used. Exported images were used to quantify relative YAP levels within each nucleus (nuclear regions traced by DAPI stained nucleus) via a custom-made MATLAB program (The MathWorks, Natick, MA). DAPI images were analyzed using an edge-detection algorithm in order to determine the regions of cell nuclei for each cell. This algorithm used a thresholding method, which defined these regions based on experimentally determined threshold parameters, identification of regions based on 8-directional connectivity of image pixels as evaluated by the “bwlabel” function, and removal of regions substantially smaller than standard nucleus size by the “bwareaopen” function. The nuclear outline was then used as a mask to quantify the average pixel intensity of the YAP stain within the individual nuclei of each cell.

Sample sizes varied between experiments based on the number of images taken. Based on power calculations with 40% effects size and standard deviation of 50%—which was our average LIV or SMG effect on YAP nuclear levels during preliminary experiments, 25 samples is sufficient to reach 80% statistical power assuming 95 percentile confidence. In each major experiment, we imaged on average 100 to 200 nuclei to quantify YAP levels, this was an approximate range as we cannot really control how many nuclei we image in each field of view during microscopy sessions. All images from the individual trials were pooled and the final sample sizes were provided in the figure legends. We have also added Supplementary Tables S4–S14 to provide exact sample sizes and analysis details.

### Atomic force microscopy

Bruker Dimension FastScan AFM was used for the collection of the atomic force measurements^81^. Tipless MLCT-D probes with a 0.03 N/m spring constant were functionalized with 10 µm diameter borosilicate glass beads for force collection. The AFM’s optical microscope was used to locate individual live MSCs plated on the SlideFlask slides with the flask section removed for access to the cells. The nucleus of each cell was tested with at least 3 s of rest between each test. In each test, three force-displacement curves were obtained (ramping rate: 2 µm/s over 2 µm total travel, 1 µm approach, 1 µm retract), which were analyzed using Nanoscope software with the implementation of a best-fit curve to a Hertzian (spherical) model (optimized such that *R*^2^ value was >0.95, or *p* < 0.05) to obtain elastic moduli of nuclear membrane of individual nuclei.

### Western blotting

Western blotting was performed using standard gel electrophoresis procedure^29,43,57,82^. 20 μg of lysed cell protein from each sample was run on 10% polyacrylamide gels, transferred onto polyvinylidene difluoride (PVDF) membranes, blocked with 5%(w/v) milk for 1 h. After washing, the membranes were incubated overnight at 4 °C in a solution of primary antibodies diluted in 5% w/v bovine serum albumin (BSA), 1X tris-buffered saline (TBS), 0.1% tween. Protein bands were visualized via horseradish peroxidase-conjugated secondary antibodies (1:5000, Cell Signaling) and ECL plus chemiluminescence kit (Amersham Biosciences, Piscataway, NJ) and scanned using C-DiGit blot scanner (Licor, Lincoln, NE). All blots derive from the same experiment and were processed in parallel.

### Statistical analysis

All data analysis results were displayed graphically based on the mean value with standard deviation. All the graphs were generated and analyzed using GraphPad Prism 8. Differences between treatments were not assumed to follow a Gaussian distribution. Therefore, group differences were identified via either non-parametric two-tailed Mann–Whitney *U*-test (Fig. 1a) or Kruskal–Wallis test followed by Tukey multiple comparisons (Figs. 2b, 4, 5, 6, 7, and Supplementary Figs. 1–3). *p*-values of < 0.05 were considered significant.

### Reporting summary

Further information on research design is available in the Nature Research Reporting Summary linked to this article.

## Supplementary information

Supplemental Material

Reporting Summary Checklist

## Data Availability

The data sets generated and/or analyzed during the current study are available from the corresponding author on reasonable request.
