# Supplementary material for: Low-intensity vibration restores nuclear YAP levels and acute YAP nuclear shuttling in mesenchymal stem cells subjected to simulated microgravity
Source: NPJ Microgravity. 2020 Dec 1;6:35. doi: 10.1038/s41526-020-00125-5 (PMC7708987; doi:10.1038/s41526-020-00125-5)
Supplement: Supplementary file 1 — Supplemental Material [file 41526_2020_125_MOESM1_ESM.pdf]

Supplementary Information

Low Intensity Vibrations Restore Nuclear YAP Levels and Acute YAP Nuclear Shuttling in Mesenchymal Stem Cells Subjected to Simulated Microgravity

Thompson M[1], Woods K[2], Newberg, J[1], Oxford JT, Uzer G[1†]

[1]Mechanical and Biomedical Engineering, Boise State University
[2]Biomolecular Sciences Graduate Program, Boise State University
† Corresponding Author

Funding support:
NASA ISGC NNX15AI04H, NIH R01AG059923, and 5P2CHD086843-03, P20GM109095, P20GM103408 and NSF 1929188.

Supplementary Figure 1.

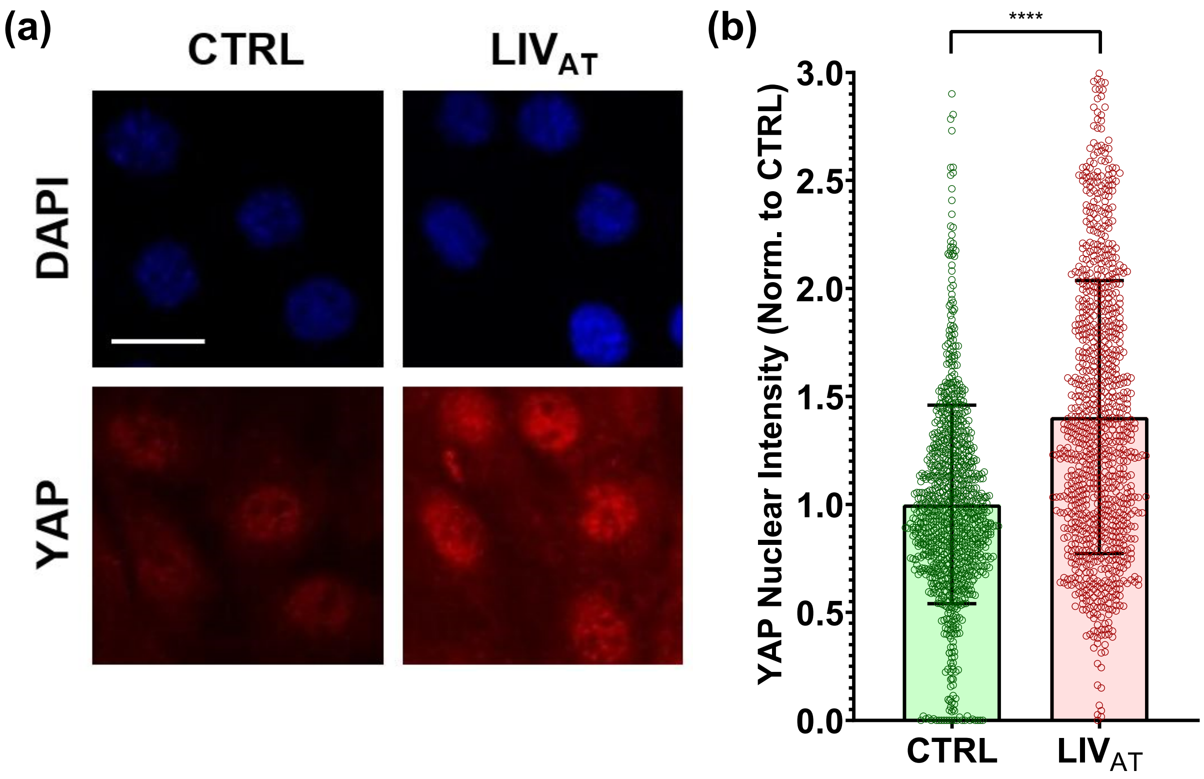

Supplementary Figure 1. LIV_{AT} treatment increases nuclear YAP in C2C12 cells. (a) C2C12 cells were subjected to LIV_{AT} and stained with DAPI (blue) and YAP (red). Confocal images displayed increased nuclear YAP levels following LIV_{AT} treatment. (b) Quantitative analysis of confocal images showed a 40% increase of nuclear YAP in LIV_{AT} samples compared to controls. n>900/grp, group comparison was made using a Mann-Whitney U-test, *p<0.05, **p<0.01, ***p<0.01, ****p<0.0001. Error bars represent standard deviation. Scale bar: 10 µm. Full statistical details were provided in Supplementary Table 12.

Supplementary Figure 2.

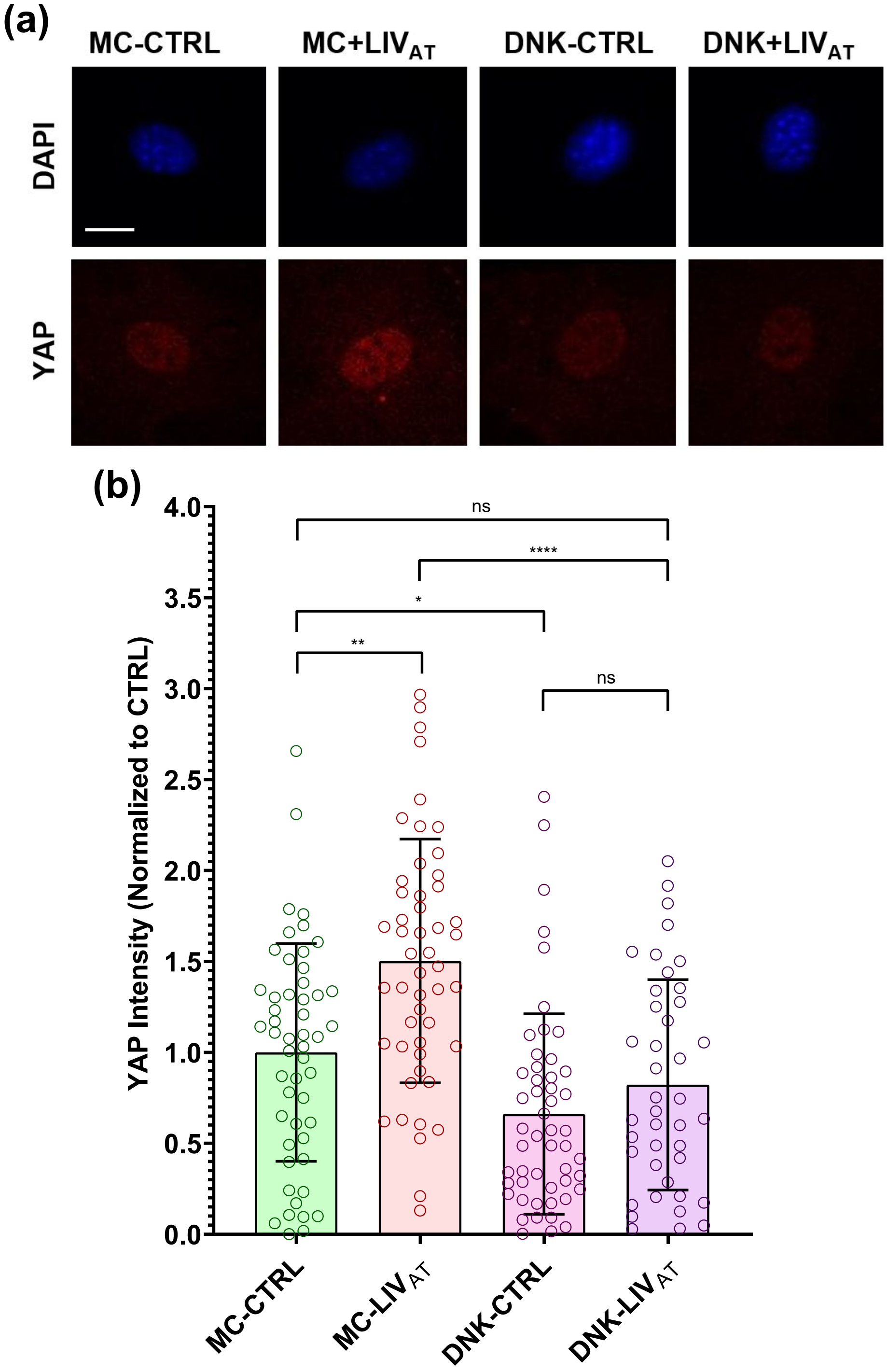

Supplementary Figure 2. LINC complex disruption decreases nuclear YAP levels and reduces LIV$_{AT}$-induced YAP nuclear entry. (a) Plasmids harboring either a dominant negative KASH domain of Nesprin (DNK) to disable LINC complex function or empty mCherry control (MC) were overexpressed in MSCs. Following puromycin selection, MC or DNK expressing MSCs were subjected to LIV$_{AT}$ and stained against DAPI (blue) and YAP (red). (b) Quantitative analysis of confocal images revealed a 49% increase of nuclear YAP following LIV$_{AT}$ in MC expressing control MSCs. Basal YAP levels of the DNK-CTRL group were 34% lower compared to MC-CTRL and LIV$_{AT}$ treatment failed to significantly increase nuclear YAP over DNK-CTRL (18%, NS). n>30/grp, group comparisons were made via Kruskal-Wallis test followed by Tukey multiple comparison, *p<0.05, **p<0.01, ***p<0.01, ****p<0.0001. Error bars represent standard deviation. Scale bar: 10 µm. Full statistical details were provided in Supplementary Table 13.

Supplementary Figure 3.

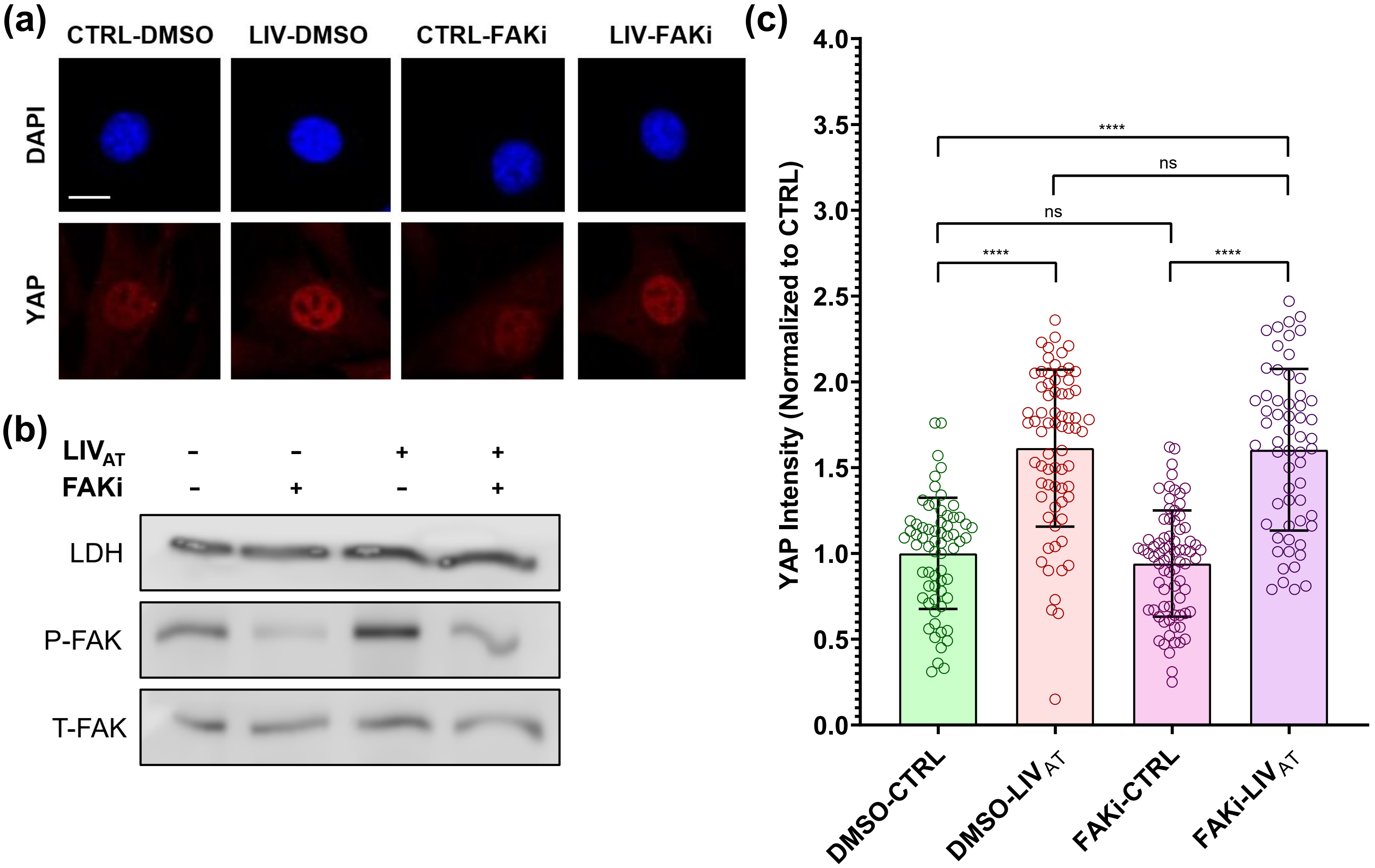

Supplementary Figure 3. Blocking FAK phosphorylation at Tyr 397 does not limit LIV_{AT} induced YAP nuclear entry. Dimethyl sulphoxide (DMSO) or Tyr 397 specific FAK inhibitor (FAKi) PF573228 (3µM) was added to MSCs in culture medium for 1h prior to LIV_{AT} or control treatments. (a) Confocal images of YAP showed more intense nuclear YAP staining of LIV_{AT} treated MSCs but no apparent effect of FAKi when compared to DMSO (b) FAKi application 1hr prior to LIV_{AT} treatment inhibited the LIV_{AT} induced FAK phosphorylation at Tyr 397 and decreased the basal levels (c) Quantitative analysis of confocal images revealed a 61% increase of nuclear YAP in both the DMSO-LIV group and a 60% increase in the FAKi-LIV_{AT} group compared to the DMSO-CTRL group. Differences between the DMSO-CTRL and FAKi-CTRL groups and between the DMSO-LIV and FAKi-LIV_{AT} groups were not significant. n>50/grp. Group comparisons were made via Kruskal-Wallis test followed by Tukey multiple comparison, $*p<0.05$, $**p<0.01$, $***p<0.01$, $****p<0.0001$. All blots were generated in the same experiment and were processed in parallel. Error bars represent standard deviation. Scale bar: 10 µm. Full statistical details were provided in Supplementary Table 14.

Supplementary Figure 4.

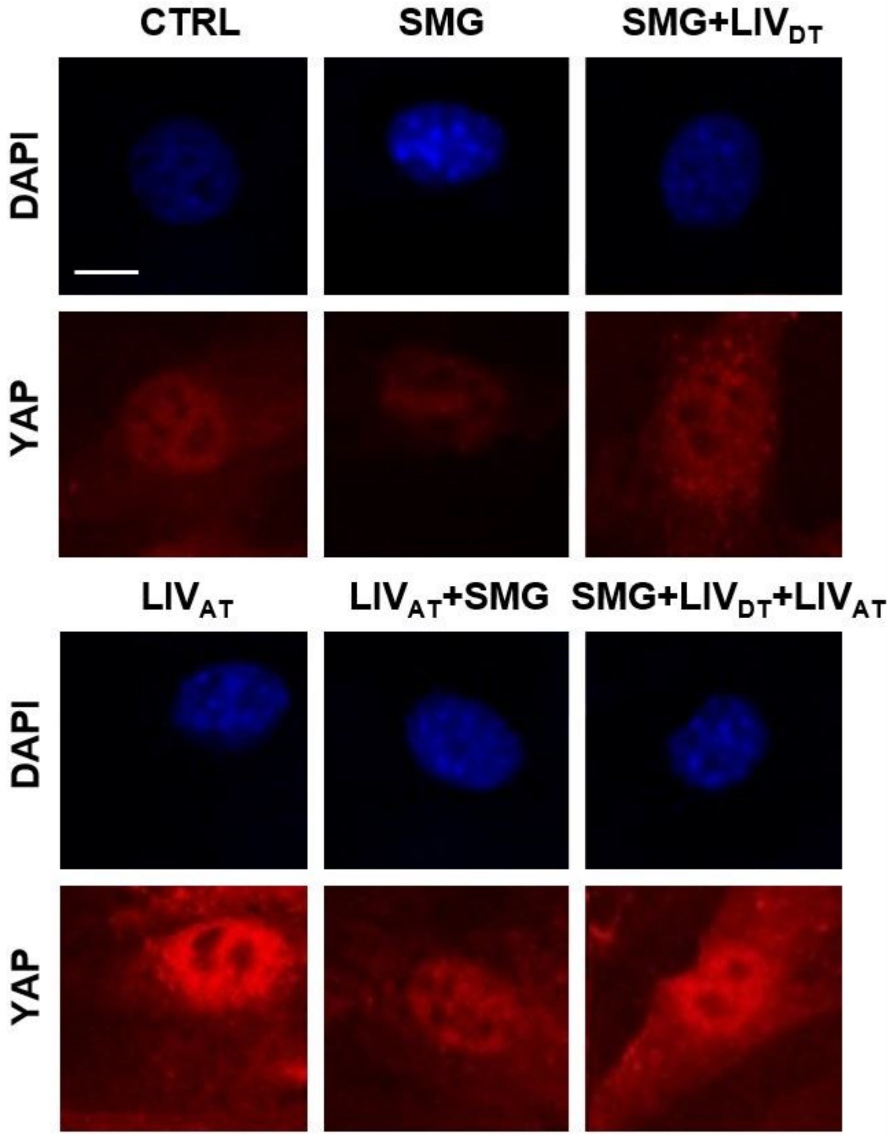

Supplementary Figure 4. Confocal images for SMG/LIV$_{DT}$/LIV$_{AT}$ treatments. Example images from the experiments represented by the data in Figure 4. Scale bar: 10 µm.

Supplementary Figure 5.

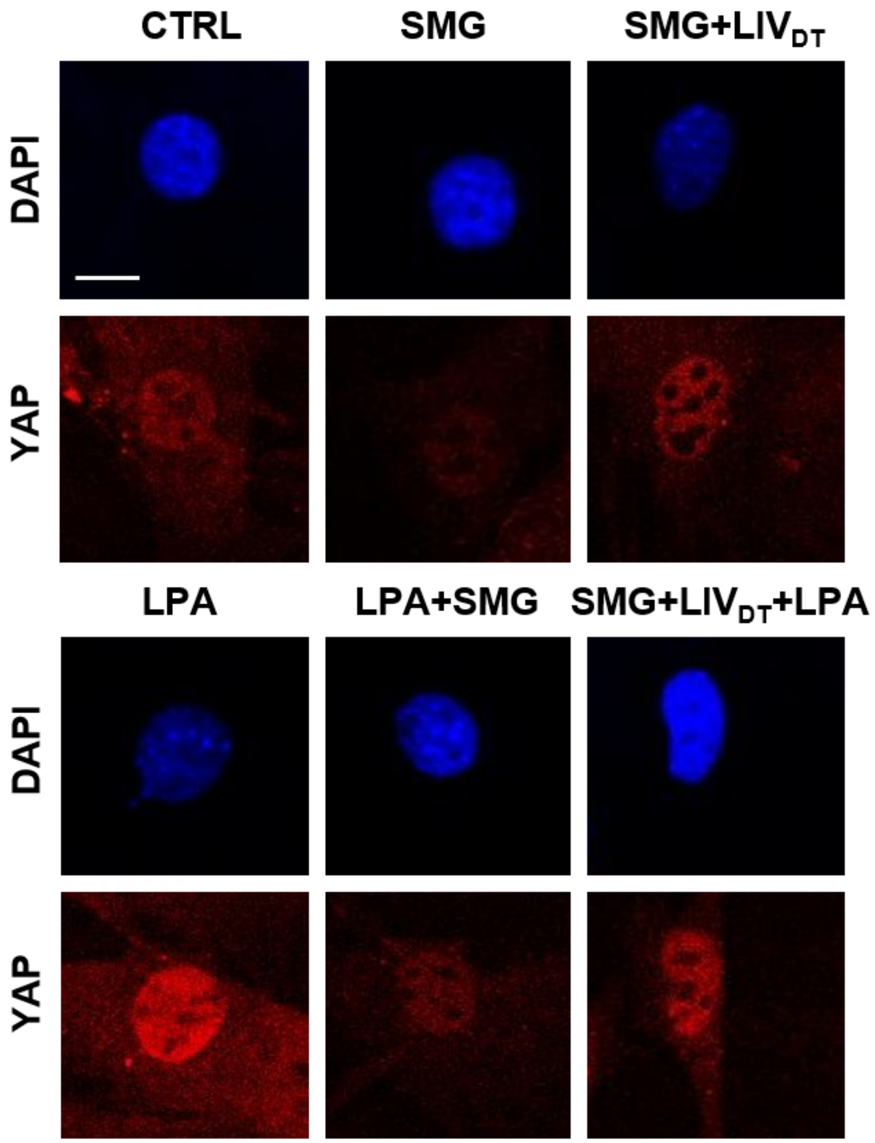

Supplementary Figure 5. Confocal images for SMG/LIV$_{DT}$/LPA treatments. Example images from the experiments represented by the data in Figure 6. Scale bar: 10 µm.

Supplementary Figure 6

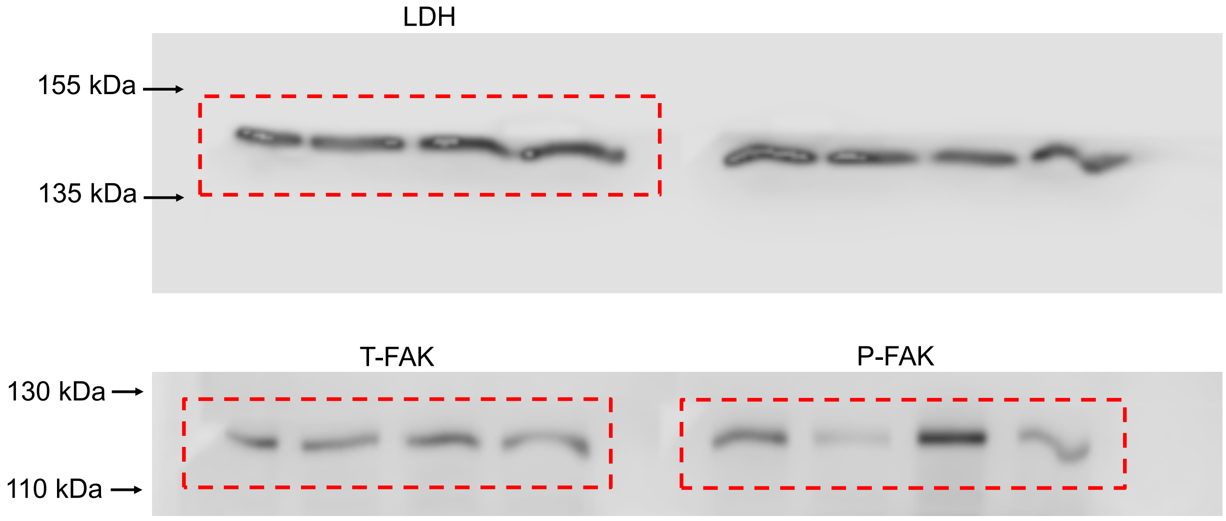

Supplementary Figure 6. Unprocessed blots for LIV$_{AT}$/FAK treatments. Uncropped Western Blot images used in Supplementary Figure 3 as obtained by LiCor C-DiGit blot scanner.

Supplementary Table 1: Cell culture and pharmacological reagents and their final concentrations.

Cell culture and pharmacological reagents		Dilution
IMDM	GIBCO	-
DMEM	Caisson Laboratories	-
FCS	Atlanta Biologicals	10% v/v
Penicillin/streptomycin	GIBCO	1% v/v

Supplementary Table 2: Antibodies used and their final concentrations for western blots.

Antibodies		Dilution
p-FAK Tyr397 (3283)	Cell Signaling Technology	1/1000
FAK (sc-558)	Santa Cruz Biotechnology	1/500
LDHA (2012S)	Cell Signaling Technology	1/1000

Supplementary Table 3: Immunostaining antibodies and reagents and their final concentrations.

Immunostaining Antibodies and Reagents		Dilution
DAPI (#H1500)	Vector Laboratories	1/1
Phalloidin-iFlour 488 Reagent (#AB17653)	Abchem	1/500
YAP (#14074)	Cell Signaling Technology	1/100
Alexa Flour 594 (#A11037)	Thermo Fisher Scientific	1/500
Alexa Flour 633 (#A21070)	Thermo Fisher Scientific	1/500

Supplementary Table 4: Mann-Whitney test statistical analysis of data in Figure 1.

Test Results	
P value	<0.0001
Exact or approximate P value?	Approximate
P value summary	****
Significantly different (P < 0.05)?	Yes
One- or two-tailed P value?	Two-tailed
Sum of ranks in CTRL	157979
Mann-Whitney U	62713
Median of CTRL	0.9607
Median of LIVAT	1.209
Median Difference: Actual	0.2478
Median Difference: Hodges-Lehmann	0.2588

Supplementary Table 5: ANOVA results for Kruskal-Wallis test for data in Figure 2.

Test Results	
P value	<0.0001
Exact or approximate P value?	Approximate
P value summary	****
Do the medians vary signif. (P < 0.05)?	Yes
Number of groups	3
Kruskal-Wallis statistic	297.3
Number of values (total)	1090

Supplementary Table 6: Multiple comparisons results for Kruskal-Wallis test for data in Figure 2.

Test Results						
Number of families	1					
Number of comparisons per family	3					
Alpha	0.05					
Dunn's multiple comparisons test	Mean rank diff.	Significant?	Summary	Adjusted P Value	Groups	
CTRL vs. SMG	360.7	Yes	****	<0.0001	A-B	
CTRL vs. SMG+LIVDT	51.38	No	ns	0.1034	A-C	
SMG vs. SMG+LIVDT	-309.3	Yes	****	<0.0001	B-C	
Test details	Mean rank 1	Mean rank 2	Mean rank diff.	n1	n2	Z
CTRL vs. SMG	688.5	327.8	360.7	417	392	16.29
CTRL vs. SMG+LIVDT	688.5	637.1	51.38	417	281	2.115
SMG vs. SMG+LIVDT	327.8	637.1	-309.3	392	281	12.57

Supplementary Table 7: ANOVA results for Kruskal-Wallis test for data in Figure 4.

Test Results	
P value	<0.0001
Exact or approximate P value?	Approximate
P value summary	****
Do the medians vary signif. (P < 0.05)?	Yes
Number of groups	6
Kruskal-Wallis statistic	530.4
Number of values (total)	1683

Supplementary Table 8: Multiple comparisons results for Kruskal-Wallis test for data in Figure 4.

Test Results						
Number of families	1					
Number of comparisons per family	15					
Alpha	0.05					
Dunn's multiple comparisons test	Mean rank diff.	Significant?	Summary	Adjusted P Value	Groups	
CTRL vs. LIVAT	-483.1	Yes	****	<0.0001	A-B	
CTRL vs. SMG	440	Yes	****	<0.0001	A-C	
CTRL vs. SMG+LIVAT	-73.34	No	ns	0.8714	A-D	
CTRL vs. SMG+LIVDT	61.18	No	ns	>0.9999	A-E	
CTRL vs. SMG+LIVDT+LIVAT	-173.9	Yes	**	0.0012	A-F	
LIVAT vs. SMG	923.1	Yes	****	<0.0001	B-C	
LIVAT vs. SMG+LIVAT	409.8	Yes	****	<0.0001	B-D	
LIVAT vs. SMG+LIVDT	544.3	Yes	****	<0.0001	B-E	
LIVAT vs. SMG+LIVDT+LIVAT	309.2	Yes	****	<0.0001	B-F	
SMG vs. SMG+LIVAT	-513.3	Yes	****	<0.0001	C-D	
SMG vs. SMG+LIVDT	-378.8	Yes	****	<0.0001	C-E	
SMG vs. SMG+LIVDT+LIVAT	-613.9	Yes	****	<0.0001	C-F	
SMG+LIVAT vs. SMG+LIVDT	134.5	Yes	**	0.0084	D-E	
SMG+LIVAT vs. SMG+LIVDT+LIVAT	-100.6	No	ns	0.2664	D-F	
SMG+LIVDT vs. SMG+LIVDT+LIVAT	-235.1	Yes	****	<0.0001	E-F	
Test details	Mean rank 1	Mean rank 2	Mean rank diff.	n1	n2	Z
CTRL vs. LIVAT	798	1281	-483.1	289	296	12.02
CTRL vs. SMG	798	358.1	440	289	259	10.58
CTRL vs. SMG+LIVAT	798	871.4	-73.34	289	347	1.895
CTRL vs. SMG+LIVDT	798	736.8	61.18	289	281	1.503
CTRL vs. SMG+LIVDT+LIVAT	798	971.9	-173.9	289	211	3.952
LIVAT vs. SMG	1281	358.1	923.1	296	259	22.32
LIVAT vs. SMG+LIVAT	1281	871.4	409.8	296	347	10.66
LIVAT vs. SMG+LIVDT	1281	736.8	544.3	296	281	13.45
LIVAT vs. SMG+LIVDT+LIVAT	1281	971.9	309.2	296	211	7.061
SMG vs. SMG+LIVAT	358.1	871.4	-513.3	259	347	12.86
SMG vs. SMG+LIVDT	358.1	736.8	-378.8	259	281	9.048
SMG vs. SMG+LIVDT+LIVAT	358.1	971.9	-613.9	259	211	13.62
SMG+LIVAT vs. SMG+LIVDT	871.4	736.8	134.5	347	281	3.449
SMG+LIVAT vs. SMG+LIVDT+LIVAT	871.4	971.9	-100.6	347	211	2.371
SMG+LIVDT vs. SMG+LIVDT+LIVAT	736.8	971.9	-235.1	281	211	5.311

Supplementary Table 9: ANOVA results for Kruskal-Wallis test for data in Figure 5.

Test Results	
P value	<0.0001
Exact or approximate P value?	Approximate
P value summary	****
Do the medians vary signif. (P < 0.05)?	Yes
Number of groups	3
Kruskal-Wallis statistic	33.43
Number of values (total)	112

Supplementary Table 10: Multiple comparisons results for Kruskal-Wallis test for data in Figure 5.

Test Results						
Number of families	1					
Number of comparisons per family	3					
Alpha	0.05					
Dunn's multiple comparisons test	Mean rank diff.	Significant?	Summary	Adjusted P Value	Groups	
CTRL vs. LPA (50uM)	-34.34	Yes	****	<0.0001	A-C	
CTRL vs. LPA (100uM)	-39.63	Yes	****	<0.0001	A-D	
LPA (50uM) vs. LPA (100uM)	-5.285	No	ns	>0.9999	C-D	
Test details	Mean rank 1	Mean rank 2	Mean rank diff.	n1	n2	Z
CTRL vs. LPA (50uM)	31.92	66.26	-34.34	38	34	4.48
CTRL vs. LPA (100uM)	31.92	71.55	-39.63	38	40	5.387
LPA (50uM) vs. LPA (100uM)	66.26	71.55	-5.285	34	40	0.6977

Supplementary Table 11: ANOVA results for Kruskal-Wallis test for data in Figure 6.

Test Results	
P value	<0.0001
Exact or approximate P value?	Approximate
P value summary	****
Do the medians vary signif. (P < 0.05)?	Yes
Number of groups	6
Kruskal-Wallis statistic	230.6
Number of values (total)	1152

Supplementary Table 12: Multiple comparisons results for Kruskal-Wallis test for data in Figure 6.

Test Results						
Number of families	1					
Number of comparisons per family	15					
Alpha	0.05					
Dunn's multiple comparisons test	Mean rank diff.	Significant?	Summary	Adjusted P Value	Groups	
CTRL vs. SMG	211.1	Yes	****	<0.0001	A-B	
CTRL vs. LPA	-305.8	Yes	****	<0.0001	A-C	
CTRL vs. SMG+LIVDT	30.58	No	ns	>0.9999	A-D	
CTRL vs. SMG+LPA	39.63	No	ns	>0.9999	A-E	
CTRL vs. SMG+LIVDT+LPA	-120.7	Yes	**	0.0056	A-F	
SMG vs. LPA	-516.9	Yes	****	<0.0001	B-C	
SMG vs. SMG+LIVDT	-180.5	Yes	****	<0.0001	B-D	
SMG vs. SMG+LPA	-171.5	Yes	****	<0.0001	B-E	
SMG vs. SMG+LIVDT+LPA	-331.8	Yes	****	<0.0001	B-F	
LPA vs. SMG+LIVDT	336.4	Yes	****	<0.0001	C-D	
LPA vs. SMG+LPA	345.4	Yes	****	<0.0001	C-E	
LPA vs. SMG+LIVDT+LPA	185.1	Yes	****	<0.0001	C-F	
SMG+LIVDT vs. SMG+LPA	9.048	No	ns	>0.9999	D-E	
SMG+LIVDT vs. SMG+LIVDT+LPA	-151.3	Yes	****	<0.0001	D-F	
SMG+LPA vs. SMG+LIVDT+LPA	-160.3	Yes	****	<0.0001	E-F	
Test details	Mean rank 1	Mean rank 2	Mean rank diff.	n1	n2	Z
CTRL vs. SMG	565.3	354.2	211.1	175	196	6.102
CTRL vs. LPA	565.3	871.1	-305.8	175	142	8.139
CTRL vs. SMG+LIVDT	565.3	534.7	30.58	175	204	0.892
CTRL vs. SMG+LPA	565.3	525.7	39.63	175	221	1.177
CTRL vs. SMG+LIVDT+LPA	565.3	686	-120.7	175	214	3.56
SMG vs. LPA	354.2	871.1	-516.9	196	142	14.1
SMG vs. SMG+LIVDT	354.2	534.7	-180.5	196	204	5.426
SMG vs. SMG+LPA	354.2	525.7	-171.5	196	221	5.254
SMG vs. SMG+LIVDT+LPA	354.2	686	-331.8	196	214	10.09
LPA vs. SMG+LIVDT	871.1	534.7	336.4	142	204	9.252
LPA vs. SMG+LPA	871.1	525.7	345.4	142	221	9.654
LPA vs. SMG+LIVDT+LPA	871.1	686	185.1	142	214	5.14
SMG+LIVDT vs. SMG+LPA	534.7	525.7	9.048	204	221	0.2801
SMG+LIVDT vs. SMG+LIVDT+LPA	534.7	686	-151.3	204	214	4.648
SMG+LPA vs. SMG+LIVDT+LPA	525.7	686	-160.3	221	214	5.025

Supplementary Table 13: ANOVA results for Kruskal-Wallis test for data in Figure 7a.

Test Results	
P value	0.8713
Exact or approximate P value?	Approximate
P value summary	ns
Do the medians vary signif. (P < 0.05)?	No
Number of groups	3
Kruskal-Wallis statistic	0.2755
Number of values (total)	30

Supplementary Table 14: Multiple comparisons results for Kruskal-Wallis test for data in Figure 7a.

Test Results						
Number of families	1					
Number of comparisons per family	3					
Alpha	0.05					
Dunn's multiple comparisons test	Mean rank diff.	Significant?	Summary	Adjusted P Value	Groups	
CTRL vs. SMG	1.25	No	ns	>0.9999	A-B	
CTRL vs. SMG+LIVDT	2.05	No	ns	>0.9999	A-C	
SMG vs. SMG+LIVDT	0.8	No	ns	>0.9999	B-C	
Test details	Mean rank 1	Mean rank 2	Mean rank diff.	n1	n2	Z
CTRL vs. SMG	16.6	15.35	1.25	10	10	0.3175
CTRL vs. SMG+LIVDT	16.6	14.55	2.05	10	10	0.5208
SMG vs. SMG+LIVDT	15.35	14.55	0.8	10	10	0.2032

Supplementary Table 15: ANOVA results for Kruskal-Wallis test for data in Figure 7c.

Test Results	
P value	0.0394
Exact or approximate P value?	Approximate
P value summary	*
Do the medians vary signif. (P < 0.05)?	Yes
Number of groups	3
Kruskal-Wallis statistic	6.468
Number of values (total)	45

Supplementary Table 16: Multiple comparisons results for Kruskal-Wallis test for data in Figure 7c.

Test Results						
Number of families	1					
Number of comparisons per family	3					
Alpha	0.05					
Dunn's multiple comparisons test	Mean rank diff.	Significant?	Summary	Adjusted P Value	Groups	
CTRL vs. SMG	11.47	No	ns	0.0504	A-B	
CTRL vs. SMG+LIVDT	9.333	No	ns	0.1549	A-D	
SMG vs. SMG+LIVDT	-2.133	No	ns	>0.9999	B-D	
Test details	Mean rank 1	Mean rank 2	Mean rank diff.	n1	n2	Z
CTRL vs. SMG	29.93	18.47	11.47	15	15	2.391
CTRL vs. SMG+LIVDT	29.93	20.6	9.333	15	15	1.946
SMG vs. SMG+LIVDT	18.47	20.6	-2.133	15	15	0.4448

Supplementary Table 17: ANOVA results for Kruskal-Wallis test for data in Figure 7d.

Test Results	
P value	0.7417
Exact or approximate P value?	Approximate
P value summary	ns
Do the medians vary signif. (P < 0.05)?	No
Number of groups	3
Kruskal-Wallis statistic	0.5977
Number of values (total)	323

Supplementary Table 18: Multiple comparisons results for Kruskal-Wallis test for data in Figure 7d.

Test Results						
Number of families	1					
Number of comparisons per family	3					
Alpha	0.05					
Dunn's multiple comparisons test	Mean rank diff.	Significant?	Summary	Adjusted P Value	Groups	
CTRL vs. SMG	2.055	No	ns	>0.9999	A-B	
CTRL vs. SMG+LIVDT	-7.273	No	ns	>0.9999	A-C	
SMG vs. SMG+LIVDT	-9.328	No	ns	>0.9999	B-C	
Test details	Mean rank 1	Mean rank 2	Mean rank diff.	n1	n2	Z
CTRL vs. SMG	160.1	158	2.055	111	99	0.1592
CTRL vs. SMG+LIVDT	160.1	167.4	-7.273	111	113	0.5828
SMG vs. SMG+LIVDT	158	167.4	-9.328	99	113	0.7257

Supplementary Table 19: Mann-Whitney test statistical analysis of data in Supplementary Figure 1.

Test Results	
P value	<0.0001
Exact or approximate P value?	Approximate
P value summary	****
Significantly different (P < 0.05)?	Yes
One- or two-tailed P value?	Two-tailed
Sum of ranks in CTRL	B
Mann-Whitney U	312590
Median of CTRL	0.9519
Median of LIVAT	1.319
Median Difference: Actual	0.3669
Median Difference: Hodges-Lehmann	0.3632

Supplementary Table 20: ANOVA results for Kruskal-Wallis test for data in Supplementary Figure 2.

Test Results	
P value	<0.0001
Exact or approximate P value?	Approximate
P value summary	****
Do the medians vary signif. (P < 0.05)?	Yes
Number of groups	4
Kruskal-Wallis statistic	42.25
Number of values (total)	193

Supplementary Table 21: Multiple comparisons results for Kruskal-Wallis test for data in Supplementary Figure 2.

Test Results						
Number of families	1					
Number of comparisons per family	6					
Alpha	0.05					
Dunn's multiple comparisons test	Mean rank diff.	Significant?	Summary	Adjusted P Value	Groups	
MC-CTRL vs. MC-LIVAT	-37.64	Yes	**	0.0043	A-B	
MC-CTRL vs. DNK-CTRL	31.92	Yes	*	0.0234	A-C	
MC-CTRL vs. DNK-LIVAT	15.42	No	ns	>0.9999	A-D	
MC-LIVAT vs. DNK-CTRL	69.56	Yes	****	<0.0001	B-C	
MC-LIVAT vs. DNK-LIVAT	53.06	Yes	****	<0.0001	B-D	
DNK-CTRL vs. DNK-LIVAT	-16.5	No	ns	0.9547	C-D	
Test details	Mean rank 1	Mean rank 2	Mean rank diff.	n1	n2	Z
MC-CTRL vs. MC-LIVAT	98.96	136.6	-37.64	51	50	3.386
MC-CTRL vs. DNK-CTRL	98.96	67.04	31.92	51	51	2.886
MC-CTRL vs. DNK-LIVAT	98.96	83.54	15.42	51	41	1.316
MC-LIVAT vs. DNK-CTRL	136.6	67.04	69.56	50	51	6.257
MC-LIVAT vs. DNK-LIVAT	136.6	83.54	53.06	50	41	4.509
DNK-CTRL vs. DNK-LIVAT	67.04	83.54	-16.5	51	41	1.408

Supplementary Table 22: ANOVA results for Kruskal-Wallis test for data in Supplementary Figure 3.

Test Results	
P value	<0.0001
Exact or approximate P value?	Approximate
P value summary	****
Do the medians vary signif. (P < 0.05)?	Yes
Number of groups	4
Kruskal-Wallis statistic	106.7
Number of values (total)	270

Supplementary Table 23: Multiple comparisons results for Kruskal-Wallis test for data in Supplementary Figure 3.

Test Results						
Number of families	1					
Number of comparisons per family	6					
Alpha	0.05					
Dunn's multiple comparisons test	Mean rank diff.	Significant?	Summary	Adjusted P Value	Groups	
DMSO-CTRL vs. DMSO-LIVAT	-92.89	Yes	****	<0.0001	A-B	
DMSO-CTRL vs. FAKi-CTRL	13.07	No	ns	>0.9999	A-C	
DMSO-CTRL vs. FAKi-LIVAT	-88.17	Yes	****	<0.0001	A-D	
DMSO-LIVAT vs. FAKi-CTRL	106	Yes	****	<0.0001	B-C	
DMSO-LIVAT vs. FAKi-LIVAT	4.721	No	ns	>0.9999	B-D	
FAKi-CTRL vs. FAKi-LIVAT	-101.2	Yes	****	<0.0001	C-D	
Test details	Mean rank 1	Mean rank 2	Mean rank diff.	n1	n2	Z
DMSO-CTRL vs. DMSO-LIVAT	95.83	188.7	-92.89	65	70	6.906
DMSO-CTRL vs. FAKi-CTRL	95.83	82.76	13.07	65	76	0.9911
DMSO-CTRL vs. FAKi-LIVAT	95.83	184	-88.17	65	59	6.28
DMSO-LIVAT vs. FAKi-CTRL	188.7	82.76	106	70	76	8.192
DMSO-LIVAT vs. FAKi-LIVAT	188.7	184	4.721	70	59	0.3421
FAKi-CTRL vs. FAKi-LIVAT	82.76	184	-101.2	76	59	7.473
