# Supplementary material for: Low-intensity vibration restores nuclear YAP levels and acute YAP nuclear shuttling in mesenchymal stem cells subjected to simulated microgravity
Source: NPJ Microgravity. 2020 Dec 1;6:35. doi: 10.1038/s41526-020-00125-5 (PMC7708987; doi:10.1038/s41526-020-00125-5)
Supplement: Supplementary file 2 — Reporting Summary Checklist [file 41526_2020_125_MOESM2_ESM.pdf]

nature research

Corresponding author(s): Gunes Uzer

Last updated by author(s): Aug 14, 2020

Reporting Summary

Nature Research wishes to improve the reproducibility of the work that we publish. This form provides structure for consistency and transparency in reporting. For further information on Nature Research policies, see our Editorial Policies and the Editorial Policy Checklist.

Statistics

For all statistical analyses, confirm that the following items are present in the figure legend, table legend, main text, or Methods section.

n/a	Confirmed	
☐	☒	The exact sample size (*n*) for each experimental group/condition, given as a discrete number and unit of measurement
☐	☒	A statement on whether measurements were taken from distinct samples or whether the same sample was measured repeatedly
☐	☐	The statistical test(s) used AND whether they are one- or two-sided<br>*Only common tests should be described solely by name; describe more complex techniques in the Methods section.*
☐	☒	A description of all covariates tested
☐	☒	A description of any assumptions or corrections, such as tests of normality and adjustment for multiple comparisons
☐	☒	A full description of the statistical parameters including central tendency (e.g. means) or other basic estimates (e.g. regression coefficient) AND variation (e.g. standard deviation) or associated estimates of uncertainty (e.g. confidence intervals)
☐	☒	For null hypothesis testing, the test statistic (e.g. *F*, *t*, *r*) with confidence intervals, effect sizes, degrees of freedom and *P* value noted<br>*Give P values as exact values whenever suitable.*
☒	☐	For Bayesian analysis, information on the choice of priors and Markov chain Monte Carlo settings
☐	☒	For hierarchical and complex designs, identification of the appropriate level for tests and full reporting of outcomes
☐	☒	Estimates of effect sizes (e.g. Cohen's *d*, Pearson's *r*), indicating how they were calculated

Our web collection on statistics for biologists contains articles on many of the points above.

Software and code

Policy information about availability of computer code

Data collection	None used
Data analysis	Used MATLAB 2019

For manuscripts utilizing custom algorithms or software that are central to the research but not yet described in published literature, software must be made available to editors and reviewers. We strongly encourage code deposition in a community repository (e.g. GitHub). See the Nature Research guidelines for submitting code & software for further information.

Data

Policy information about availability of data

All manuscripts must include a data availability statement. This statement should provide the following information, where applicable:

- Accession codes, unique identifiers, or web links for publicly available datasets
- A list of figures that have associated raw data
- A description of any restrictions on data availability

The datasets generated and/or analyzed during the current study are available from the corresponding author on reasonable request.

Field-specific reporting

Please select the one below that is the best fit for your research. If you are not sure, read the appropriate sections before making your selection.

☒ Life sciences ☐ Behavioural & social sciences ☐ Ecological, evolutionary & environmental sciences

For a reference copy of the document with all sections, see nature.com/documents/nr-reporting-summary-flat.pdf

Life sciences study design

All studies must disclose on these points even when the disclosure is negative.

Sample size	Sample sizes were determined via power calculators using estimated differences and data variability based on ourpreliminary data to reach 80% power. We did not perform any predetermined methods to assign sample before starting the sMG experiments but used our preliminary data to determine required sample size via power calculators using estimated differences and data variability based on our preliminary testing.
Data exclusions	No data exclusion was performed.
Replication	All data had three technical replicates and experiments independently repeated at least three times.
Randomization	When samples were assigned randomly to each group for each experiment during the start, researcher became aware of the group assignments as they had to treat them for sMG and or LIV.
Blinding	As same researchers did the data analysis they were not blinded.

Reporting for specific materials, systems and methods

We require information from authors about some types of materials, experimental systems and methods used in many studies. Here, indicate whether each material, system or method listed is relevant to your study. If you are not sure if a list item applies to your research, read the appropriate section before selecting a response.

Materials & experimental systems

n/a	Involved in the study	
☐	☒	Antibodies
☐	☒	Eukaryotic cell lines
☒	☐	Palaeontology and archaeology
☒	☐	Animals and other organisms
☒	☐	Human research participants
☒	☐	Clinical data
☒	☐	Dual use research of concern

Methods

n/a	Involved in the study	
☒	☐	ChIP-seq
☒	☐	Flow cytometry
☒	☐	MRI-based neuroimaging

Antibodies

Antibodies used	p-FAK Tyr397 (3283) Cell Signaling 1/1000<br>FAK (sc-558) Santa Cruz Biotechnology 1/500<br>LDHA (2012S) Cell Signaling Technology 1/1000
Validation	All antibodies used were validated by the companies<br>p-FAK Tyr397 (328 3) Cell Signaling - Phospho-FAK (Tyr397) Antibody detects endogenous levels of FAK only when phosphorylated at Tyr397. This antibody may cross-reacts with other tyrosine-phosphorylated RTKs. Species Reactivity: Human, Mouse, Rat, Hamster, Pig<br>FAK (sc-558) Santa Cruz Biotechnology 1/500- FAK (C-20) is recommended fordetection of FAKp125 and FRNKp4 of mouse,rat , human,chicken,Xenopuslaevisandzebrafish origin by Western Blotting.<br><br>LDHA (2012S) Cell Signaling Technology 1/1000 -LDHA Antibody detects endogenous levels of total LDHA protein. Human, Mouse, Rat, Monkey

Eukaryotic cell lines

Policy information about cell lines

Cell line source(s)	Primary MSCs
Authentication	Cells were checked for differentiation potential.
Mycoplasma contamination	No mycoplasma contamination was detected.
Commonly misidentified lines (See ICLAC register)	N/A
